# Response to Correspondence: Hajnal et al. ‘The role of lin-12 notch in *C. elegans* anchor cell proliferation’

**DOI:** 10.1242/bio.061817

**Published:** 2024-12-30

**Authors:** Michael A. Q. Martinez, David Q. Matus

**Affiliations:** 1 Department of Biochemistry and Cell Biology, Stony Brook University, Stony Brook, NY 11794-5215, USA.; 2 Present address: Department of Molecular and Cell Biology, University of California Berkeley, Berkeley, CA 94720-3200, USA.

The Correspondence by Hanjal et al. (2024) addresses the mitogenic role of *lin-12* (Notch) downstream of *egl-43* depletion in the *C. elegans* anchor cell (AC). The authors originally demonstrated that dual RNAi depletion of *egl-43* and *lin-12* reduces the *egl-43(RNAi)*-induced AC proliferation phenotype (Deng et al., 2020). In a follow-up study, we used an orthogonal approach to functionally degrade LIN-12 using the auxin-inducible degron (AID) system and found that while degradation of LIN-12 phenocopies the null allele for AC/VU fate specification, it did not rescue the *egl-43(RNAi)-*induced AC proliferation phenotype (Martinez et al., 2022). As part of our experimental workflow, we included quantitative measurements of cell cycle state using a ratiometric CDK activity sensor (Adikes et al., 2020; Martinez and Matus, 2022; Spencer et al., 2013), as the AC normally exists in a CDK-low G0 state (Smith et al., 2022). By pairing our degradation experiments with CDK activity readouts, we concluded that LIN-12 degradation in an *egl-43(RNAi)* background had no effect on the AC proliferation phenotype induced by *egl-43* depletion (Martinez et al., 2022).

Hajnal et al. (2024) paired the *lin-12* null allele with *egl-43(RNAi)* and demonstrate a significant reduction in the number of ACs observed compared to *egl-43(RNAi)* alone. This result supports their original hypothesis that *lin-12* ectopically functions as a mitogen downstream of *egl-43* depletion (Deng et al., 2020). However, we still have several questions, particularly regarding the penetrance of proliferation rescue. Why does the strongest rescue of the *egl-43(RNAi)*-induced AC proliferation phenotype come from the double RNAi experiment (*egl-43i+lin-12i*: 1.244 ACs/animal), compared to the *lin-12* null allele+*egl-43(RNAi)* (1.686 ACs/animal)? We would have expected the null allele+RNAi to generate the strongest rescue, but this might reflect the general difficulty of interpreting double RNAi experiments. These experiments involve mixing two bacterial strains expressing the target RNAi vectors, which have been shown to yield poor results (Min et al., 2010). It was the use of double RNAi to deplete LIN-12 and EGL-43 that motivated the orthogonal approach we used in our follow-up study.

Additionally, we find the logic presented in the Correspondence unclear regarding how auxin-induced degradation can phenocopy the null allele for the AC/VU decision but fail to sufficiently deplete the ectopic LIN-12 induced by *egl-43(RNAi)* to rescue AC proliferation. Our *lin-12::mNG::AID* allele allows us to visualize fluorescence following auxin treatment, and we detected no residual LIN-12 signal following auxin treatment (Martinez et al., 2022). However, we acknowledge that biologically relevant amounts may still be present, even if undetectable to the eye. Nonetheless, further experimentation is needed to determine the dose of LIN-12 required for both VU fate specification during the AC/VU decision and *egl-43(RNAi)*-induced AC proliferation.

Finally, if LIN-12 were functioning ectopically as a mitogen, the most likely mechanism would involve its entry into the nucleus to transcriptionally control downstream cell cycle targets. We recognize that there are cleavage-independent mechanisms for Notch function, however, the original observation that Notch was functioning as a mitogen in the AC was through overexpression of the NICD via a transgene (Deng et al., 2020). The series of endogenously-tagged *lin-12* alleles we generated (Medwig-Kinney et al., 2022; Pani et al., 2022) allow us to visualize LIN-12::mNG nuclear localization, which we can use to correctly identify the AC/VU decision (Medwig-Kinney et al., 2022), as LIN-12::mNG localizes to the nucleus in the VU-fated cell. While we can confirm that loss of the transcription factors *egl-43* and *nhr-67* induce AC proliferation phenotypes and generate ectopic *lin-12::mNG* expression, we have never observed LIN-12::mNG::AID signal in the nucleus of proliferating ACs, where we would expect to see it if LIN-12 were functioning to activate Notch targets (Martinez et al., 2022; Medwig-Kinney et al., 2023). However, we acknowledge that we do not know the amount of nuclear NICD that might be required to induce cell cycle entry, and it could be below the detection threshold of our EM-CCD camera.

Perhaps the most definitive experimental approach to examine whether LIN-12 is functioning as an ectopic mitogen in the *egl-43* pathway would be to generate a conditional allele of *egl-43*, either by Cre/Lox or FLP/FRT (Spiri et al., 2022) in the *lin-12(0)* background.

The cell cycle control mechanism in the invasive *C. elegans* AC is a powerful paradigm for understanding the connections between cell fate, cell cycle state, and the underlying cell biology following these transitions between proliferative and invasive fates. We are thankful to Biology Open for providing a public forum to explore this question thoroughly and would like to commend the authors on pursuing this additional challenging experiment given our previous conflicting results.
